# Simultaneous obstruction of all four bridging stents after branched endovascular repair with urgent visceral debranching as salvation therapy: A case report

**DOI:** 10.1016/j.xjse.2025.100050

**Published:** 2025-03-26

**Authors:** Konstantinos-Eleftherios Koumarelas, Drosos Kotelis, Vladimir Makaloski

**Affiliations:** Department of Vascular Surgery, Inselspital, Bern University Hospital, University of Bern, Bern, Switzerland

**Figure:**
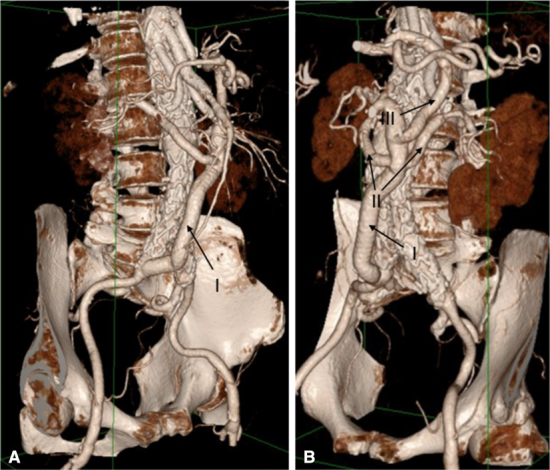
Postlaparotomy iliac-SMA bypass.

**Central Message**

F/BEVAR is effective for TAAA repair, yet bridging stent occlusions can occur. This case highlights the need for vigilant follow-up and tailored antithrombotic therapy to optimize outcomes.

Fenestrated/branched endovascular aneurysm repair (F/BEVAR) is the first line of treatment of thoracoabdominal aortic aneurysm (TAAA) in high-risk patients.^1^^,^^2^ In TAAA cases, midterm outcomes, including mortality and paraplegia, are in favor of endovascular approaches.^1^ Branched endovascular aneurysm repair (BEVAR) offers low early mortality and good survival. However, up to 30% of patients may need secondary interventions, most of them related to the bridging stents.^3^ Although initial patency rates of bridging stents are promising, the freedom from target vessel occlusion decreases during follow-up, thus explaining the importance of anticoagulation or antiplatelet therapy and understanding bridging stents’ mechanical features.^3^, ^4^, ^5^^,^E1, E2, E3

## Case Presentation

A 57-year-old-patient presented for a scheduled follow-up examination after replacement of the supracoronary ascending aorta and the entire aortic arch with frozen elephant trunk. The computed tomography (CT) angiography revealed in a period of only 2 months a significant increase of the previously known postdissection thoracoabdominal aortic aneurysm type III, according to the modified Crawford classification, from 55 mm to 66 mm at the infrarenal segment and a progressive thrombosis of the false lumen (Video 1). Given these factors, along with the symptomatic postdissection aneurysm (dull retrosternal and back pain), an urgent and complex endovascular repair was planned, performing a thoracic endovascular aortic repair, a BEVAR using an off-the-shelf multibranch stent graft, and a distal extension with endovascular aneurysm repair under general anesthesia. Our goal was to depressurize the aneurysm sac through complete thoracoabdominal aortic repair, with temporary sac perfusion maintained via the right renal artery branch, instead of staging the repair and leaving the infrarenal portion for a second intervention, which would carry a risk of rupture between procedures. The operation proceeded uneventfully, with 3 branches successfully bridged to their target vessels, the celiac trunk, superior mesenteric artery (SMA), and left renal artery. The fourth branch, directed to the right renal artery, was intentionally left patent to preserve antegrade perfusion to several segmental arteries, thereby reducing the risk of paraplegia. Because of personal issues, the patient declined early completion, and the final stage of the procedure was performed 1 month postdischarge. Because of a narrow aortic diameter at the level of the right renal artery and its steep take-off, the catheterization was challenging, creating intraoperatively a dissection of the artery, which was successfully resolved. Routine postoperative CT angiography demonstrated a good result with preserved perfusion of all reno-visceral arteries and complete exclusion of the aneurysm without any endoleak (Video 2). The bridging stent E-Ventus by JOTEC (balloon-expandable and covered) with relining using EverFlex by Medtronic (self-expanding and uncovered) was used for all vessels. We discharged the patient in good general condition with 20 mg of rivaroxaban and 100 mg of acetylsalicylic acid on a daily basis because of his previously known atrial fibrillation.

**Figure 1:**
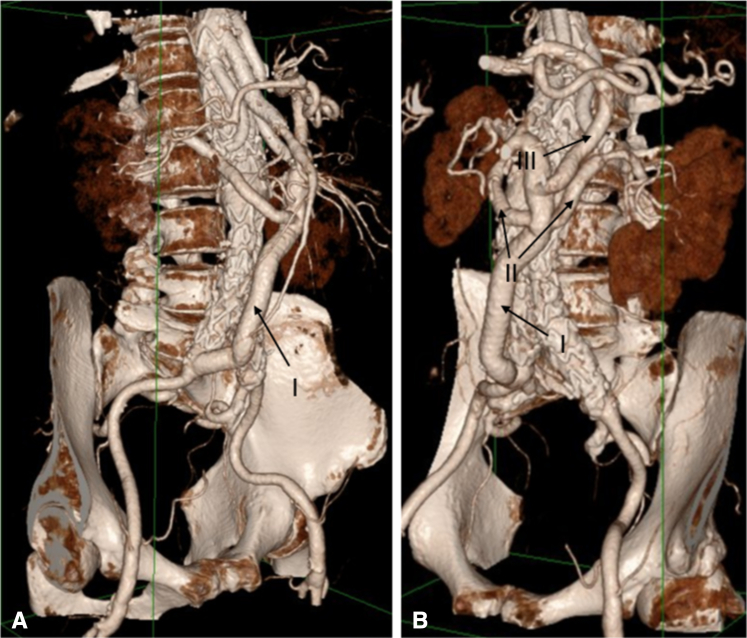
A, Ilio-SMA bypass (I), with side branches (B) to renal arteries (II) and bypass to celiac tripod (III).

**Figure 2:**
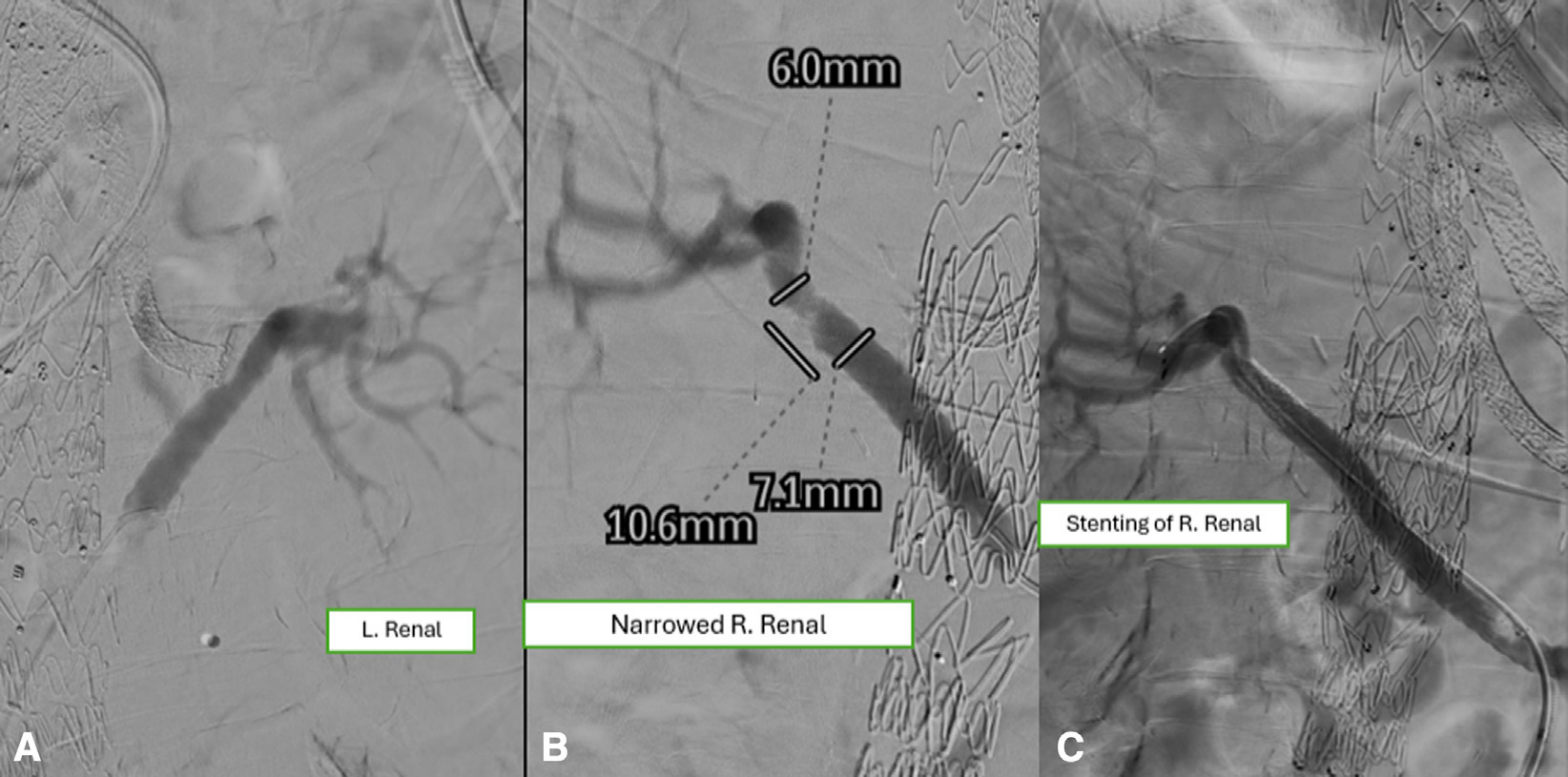
Completed and patent branches in the DSA. Patent left renal artery (A), narrowing of right renal artery (B), and stenting of right renal artery (C).

One month later, the patient experienced acute and severe abdominal pain, which led to immediate referral to the emergency department. Before the onset of pain, the patient had normal diuresis, which ceased once the pain began. After the patient's arrival, immediate thoracoabdominal CT angiography was conducted, which revealed total occlusion of all 4 BEVAR branches and severe renal and visceral ischemia (Video 3). Except for the hemodynamically not relevant known stenosis at the celiac trunk due to the arcuate ligament and the small dissection membrane or residual thrombus distally from the bridging stent in the right renal artery, no other issues had been observed in the branches, bridging stents, and target vessels. The patient was compliant, took his anticoagulation therapy regularly, and reported a moderate gastrointestinal infection 2 days before the admission, which resolved spontaneously. Emergency revascularization was clearly indicated for vital reasons, and the patient agreed to proceed. Given the simultaneous occlusion of 4 critical branches (renal, accessory renal, hepatic, and superior mesenteric arteries) and the already compromised condition of the kidneys, we chose open surgery to maximize direct access, precision, and control over blood flow restoration. This approach enhances the ability to address complex occlusions, minimize the risk of further vessel compromise, and manage potential complications more effectively compared with minimally invasive techniques, particularly in cases with multiple vessel involvement and high-stakes vascular anatomy. An emergency laparotomy with an iliac-mesenteric 10-mm Dacron bypass (from the right common iliac artery to the SMA) with additional 8-mm grafts to both renal arteries was performed (Figure 1). No intestinal resection was necessary. Early postoperatively, he required hemodialysis, and a duplex ultrasound of both kidneys demonstrated diminished perfusion of the right side. A percutaneous transluminal angioplasty with stenting of the right renal artery anastomosis correcting relevant stenosis was performed (Figure 2). In the following days, an increase in the transaminase level and suspected gastric mucosal ischemia were observed. The liver ultrasound demonstrated reduced arterial perfusion, which indicated an additional revascularization of the celiac trunk. During the emergency visceral debranching, intraoperative ultrasound assessment of hepatic perfusion after the bypass to the SMA demonstrated sufficient liver perfusion, rendering the hepatic bypass unnecessary at that time. A revision laparotomy was performed on postoperative day 16, and the revascularization of the celiac trunk was achieved from the previous prosthetic iliac-SMA bypass to the common hepatic artery with an additional 8-mm polytetrafluoroethylene bypass, routed retro-pancreatically. After a gradual improvement in enteral intake, parenteral nutrition was discontinued. The patient was treated for a *Clostridium difficile* infection with antibiotics for 2 weeks. After temporary hemodialysis, the dialysis catheter was removed on day 22 after debranching. Because of persistent paralytic ileus, nausea, vomiting, and abdominal pain, advancing the diet was difficult, and upon transfer to the ward, enteral nutrition was stopped. A nasogastric tube was placed, parenteral nutrition was restarted, and bowel-stimulating therapy with Primperan, Neostigmine, and Telebrix was initiated. Parenteral nutrition was used early postoperatively for 3 days in total, after which we continuously reduced this and switched completely to enteral nutrition on the discharge day. He was discharged on postoperative day 43 without hemodialysis and normal bowel function. The patient was followed with annual surveillance, and after 3.5 years of follow-up all visceral bypasses remained patent and the postdissection aneurysm was stable. The Institutional Review Board of the University Hospital of Bern approved the study protocol and publication of data. The patient provided informed written consent for the publication of the study data.

## Discussion

After experiencing an acute type A dissection, our patient experienced rapid increase of the postdissection thoracoabdominal aneurysm. Despite the successful early postoperative course, a subsequent renal and visceral ischemia was due to the occlusion of all 4 BEVAR branches. The revascularization was performed, including multiple bypass grafts to the mesenteric and renal arteries. In the postoperative period, complications arose, including acute renal failure, ischemic gastritis, and prolonged gastroparesis, necessitating further interventions. Despite challenges, revascularization of the celiac trunk and intensive postoperative care led to gradual improvement in visceral perfusion and enteral nutrition, although recovery remained complicated by persistent gastrointestinal issues and a focal hepatic infarct. After 41 months of follow-up, the patient did not experience any health problems related to the occlusions or surgeries.

Occlusion of bridging stents after BEVAR is a major concern, with a mean time to occlusion of 16.5 months, and thus a postoperative antithrombotic regimen over a prolonged period is important.^E4^ The European Society for Vascular Surgery supports the use of antiplatelet medication after any kind of operation for an abdominal aortic aneurysm with a B level of recommendation.^5^ In this case, the patient was receiving a dual anticoagulative regimen with aspirin and rivaroxaban, and despite that a synchronous obstruction of all 4 branches was observed. An effort was made to identify potential causative factors. The patient had recovered well from a COVID-19 infection 3 months before the occlusion. Dehydration from a recent gastrointestinal infection was also noted.^E5^^,^^E6^ Autoantibodies and genetic tests for vasculitis and thrombogenic risk were performed. It remains unclear whether the occlusion resulted from mechanical or hematological factors. Despite significant advances in branched endovascular aortic repair, the target vessel stent occlusion remains a critical concern. Overall, renal artery occlusions tend to be more common, with rates as high as 10% being reported, particularly when multiple stents or different stent types are used on the same vessel, increasing the risk of stent fracture and subsequent occlusion.^E5^ Additionally, extensive branch length and increased vessel tortuosity have been associated with greater instability and a higher likelihood of occlusion.^E5^ However, the outcomes vary. Migliari and colleagues^E7^ observed that of 345 target vessels treated in 106 patients, only 12 occlusions were observed, indicating a low incidence but highlighting the issue of long-term durability. Similar results were reported by Chen and colleagues^E8^ in patients with residual aortic dissection, with 1 of 33 patients experiencing an occlusion in a branched visceral artery after endovascular repair.^E9^ In an observational study, Gallitto and colleagues^E10^ reported the occurrence of 15 target vessel occlusions in 197 patients (renal arteries, 10; SMA, 3; celiac trunk, 2).^E7^ In their single-center analysis of 433 F/BEVAR patients with 1648 target vessels, Gomes and colleagues^E4^ reported 24 target vessels occlusions in 20 patients.^E4^ An analysis by Gorgatti and colleagues^4^ reported 9 target vessel occlusions in patients who underwent F/BEVAR with an inner branched device. During the follow-up of 24 months, 9% of the patients experienced target vessel stenosis and occlusions. Dabravolskaite and colleagues^E9^ observed that the occlusion rates of target visceral vessel at 5 years were 10.6% (5.6%-15.7%) for renal branches and 3.7% (0.7%-6.8%) for visceral branches.^E9^ Despite all these results reported in the literature, there are no current clear recommendations for the use of antithrombotic therapy postoperatively.^E5^ According to the PRINCE2SS study, resuming to aspirin within 24 hours post-F/BEVAR and to P2Y12 inhibitors such as clopidogrel within 24 to 48 hours after any spinal cord ischemia concerns are recommended. If cerebrospinal fluid drainage is placed, P2Y12 inhibitors should resume 24 hours after removal. For long-term follow-up, single antiplatelet therapy with aspirin is generally advised, even alongside oral anticoagulants. Although dual antiplatelet therapy may be beneficial for 1 to 6 months after surgery, lifelong dual antiplatelet therapy may be necessary in complex cases. For patients with unexplained target vessel occlusions, a more aggressive antithrombotic approach may be required. Each case should be reevaluated by a multidisciplinary team, especially if severe bleeding occurs.^E11^

Occlusion rates are marginal, and the simultaneous occlusion of 2 branches are rare. However, in this case we presented the critical condition of the occlusion of all 4 branches at the same time. This requires urgent intervention and revascularization of the occluded branches to avoid complete ischemia and necrosis of the tissues. Visceral debranching and ilio-SMA bypass via laparotomy are beneficial with a good long-term outcome.

## Conclusions

Ultimately, although the overall success rates of BEVAR and F/BEVAR are promising, occlusion of arterial branches remains a challenge, necessitating ongoing research and careful postoperative management to improve patient outcomes.

## Conflict of Interest Statement

D.K. and V.M. report research and educational grants to the institution from Abbott, Artivion, Biotronik, Boston Scientific, Cook, Medtronic, Nexamedic, Shockwave, and Terumo Aortic. V.M. works as consultant and proctor for Artivion. The remaining author reported no conflicts of interest.

The *Journal* policy requires editors and reviewers to disclose conflicts of interest and to decline handling or reviewing manuscripts for which they may have a conflict of interest. The editors and reviewers of this article have no conflicts of interest.

## Supplementary Data

Video 1 Postdissection type 3 thoracoabdominal aneurysm. Video available at: https://www.jtcvs.org/article/S2950-6050(25)00009-9/fulltext.

Video 2 Completion of BEVAR, with all 4 patent vessels and occlusion of the aneurysm without any endoleak. Video available at: https://www.jtcvs.org/article/S2950-6050(25)00009-9/fulltext.

Video 3 Postoperative CT, after visceral debranching, simultaneously occluded all 4 BEVAR branches and ilio-SMA bypass. Video available at: https://www.jtcvs.org/article/S2950-6050(25)00009-9/fulltext.
