# Supplementary figures and images for: Simultaneous obstruction of all four bridging stents after branched endovascular repair with urgent visceral debranching as salvation therapy: A case report
Source: JTCVS Struct Endovasc. 2025 Mar 26;7:100050. doi: 10.1016/j.xjse.2025.100050 (PMC13244756; doi:10.1016/j.xjse.2025.100050)

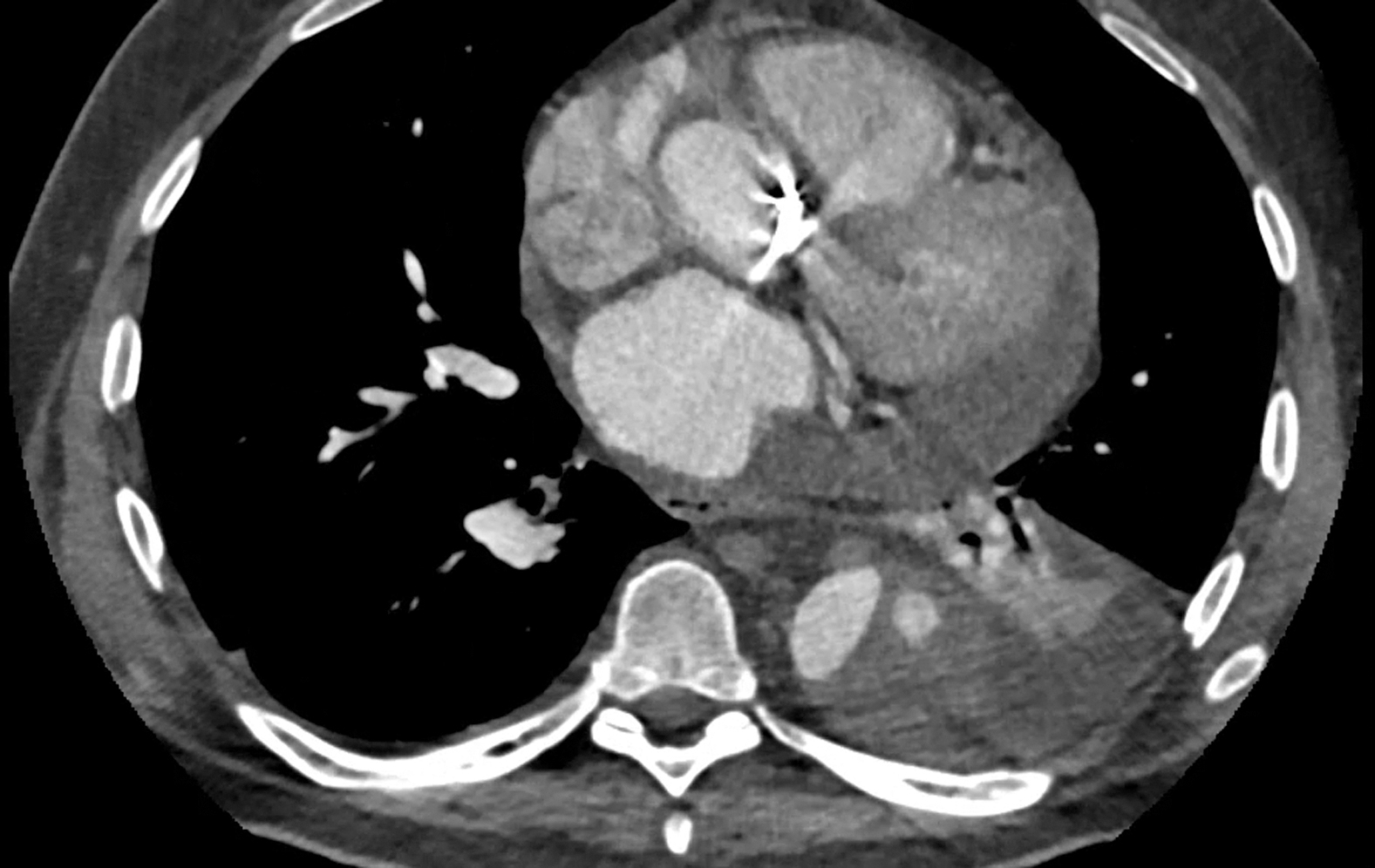

Supplement: Video 1 — Postdissection type 3 thoracoabdominal aneurysm. Video available at: https://www.jtcvs.org/article/S2950-6050(25)00009-9/fulltext. [file fx2.jpg]

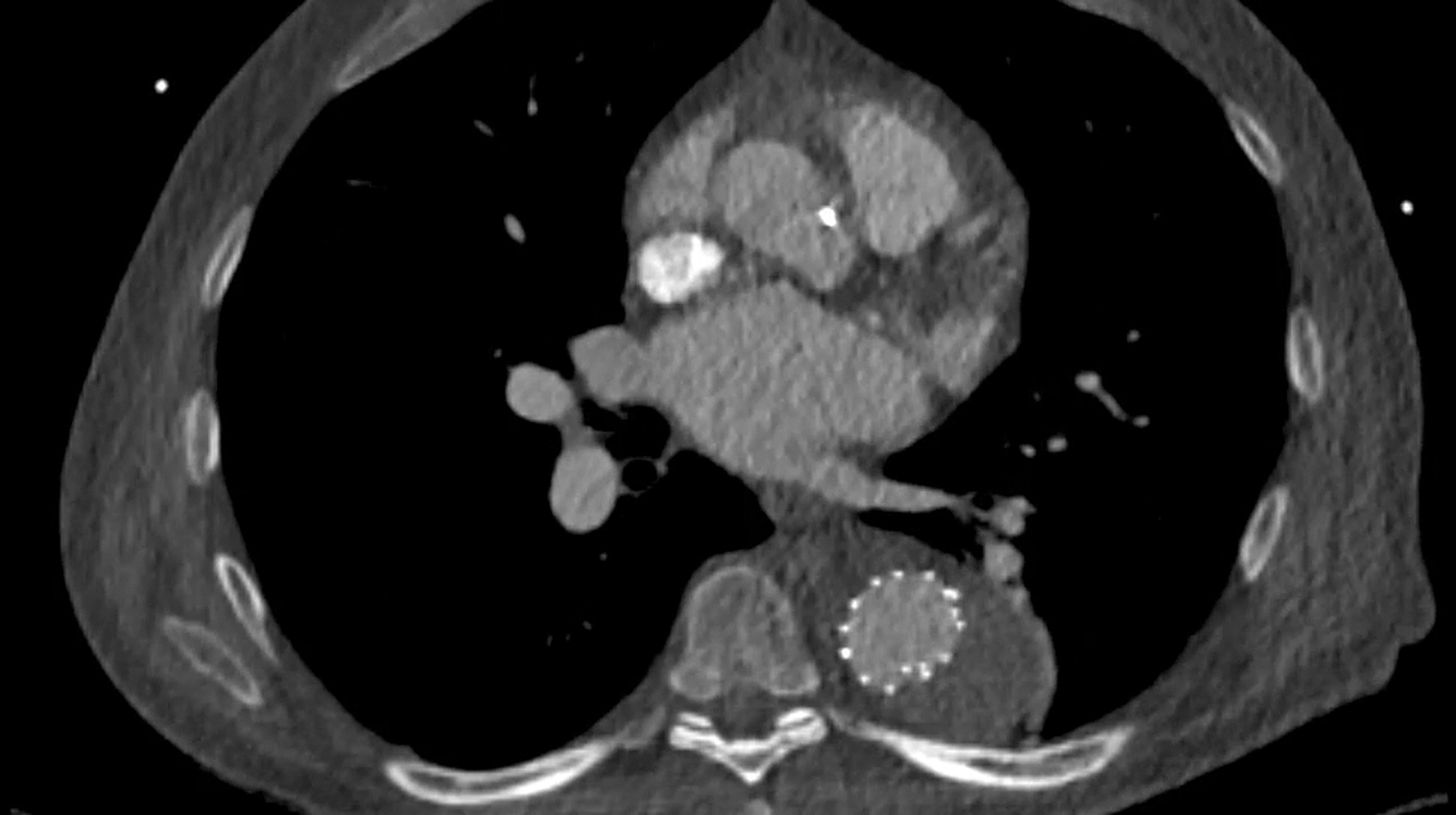

Supplement: Video 2 — Completion of BEVAR, with all 4 patent vessels and occlusion of the aneurysm without any endoleak. Video available at: https://www.jtcvs.org/article/S2950-6050(25)00009-9/fulltext. [file fx3.jpg]

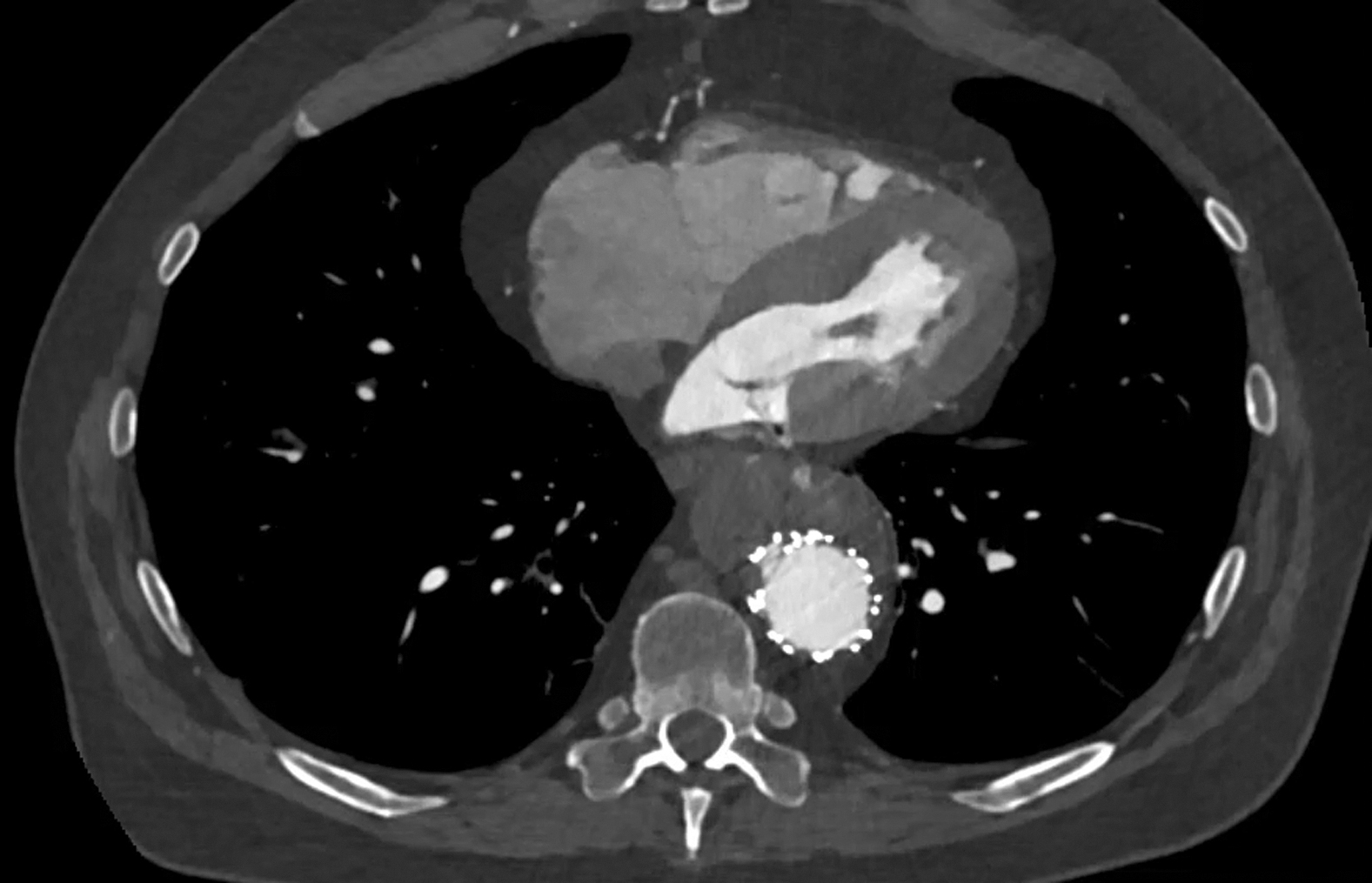

Supplement: Video 3 — Postoperative CT, after visceral debranching, simultaneously occluded all 4 BEVAR branches and ilio-SMA bypass. Video available at: https://www.jtcvs.org/article/S2950-6050(25)00009-9/fulltext. [file fx4.jpg]
